# A new isopod species of the genus *Edotia* (Valvifera, Idoteidae) from Argentina, with a key to South American species

**DOI:** 10.3897/zookeys.1267.174707

**Published:** 2026-01-30

**Authors:** Julián Santiago, Emanuel Pereira

**Affiliations:** 1 Universidad de Buenos Aires, Facultad de Ciencias Exactas y Naturales, Departamento de Biodiversidad y Biología Experimental (DBBE), Buenos Aires, Argentina Universidad de Buenos Aires Buenos Aires Argentina; 2 Universidad de Buenos Aires – CONICET, Instituto de Biodiversidad y Biología Experimental y Aplicada (IBBEA), Buenos Aires, Argentina Universidad de Buenos Aires - CONICET Buenos Aires Argentina

**Keywords:** Argentina, *Edotia dotiae* sp. nov., identification key, new species, Patagonia, Southwest Atlantic, taxonomy, valviferan isopod

## Abstract

A new valviferan isopod—*Edotia dotiae***sp. nov**.— is described from the shallow coastal waters of Argentine Patagonia. The specimens were collected during three different expeditions in the Chubut and Santa Cruz provinces and in the Beagle Channel. The new species is distinguished from other *Edotia* species by the presence of two dorsal lobes on the head, a distally protruding pleotelson and uropods with elongated endopods reaching the tip of the pleotelson. In addition, it displays discrete patches of fluted tubercles on its dorsal and lateral body surface. These structures are described in detail based on SEM images. An identification key for *Edotia* species from South America is presented.

## Introduction

The valviferan isopod genus *Edotia* Guérin-Méneville, 1843 currently comprises 20 species (Boyko et al. 2025) distributed along both coasts of North and South America, in the Atlantic sector of the Subantarctic region, and in the Weddell and Ross Sea, Antarctica (Richardson 1905; Menzies and Barnard 1959; Schultz 1969; Brusca and Wallerstein 1979; Menzies and Kruczynski 1983; Müller 1988; Brandt 1990; Wägele 1991; Castelló 2004; Brandt and Bruce 2006; Stebbins and Wetzer 2023). This genus reaches its highest diversity (nine species) in the temperate and cold waters of the Southwest Atlantic Ocean (see Pereira and Doti 2017 and references therein). Regarding their bathymetric distribution, 10 species occur in shallow waters (≤ 100 m depth; Richardson 1905; Menzies 1962; Kussakin 1982; Müller 1988; Wägele 1991; Schotte et al. 2009; Pereira 2017), eight species reach depths between 180–500 m (Kussakin 1982; Brandt 1990; Wägele 1991; Brandt and Bruce 2006; Pereira 2017), and the blind species *Edotia abyssalis* Pereira & Doti, 2017 was recorded at 2950–3282 m depth in the Mar del Plata submarine canyon (Pereira and Doti 2017; Pereira et al. 2025). The species *Edotia lilljeborgi* Ohlin, 1901 has no depth records (Ohlin 1901).

Most species of *Edotia* are small and oval, broadest at pereonites 3–5, and they display relatively smooth body surfaces. The primary exceptions are *Edotia pulchra* Brandt, 1990 with tubercles in the posterior part of the pleotelson, and *E. tangaroa* Brandt & Bruce, 2006 with cauliflower-shaped tubercles on the head and small blunt tubercles on the dorsal body surface (Brandt 1990; Brandt and Bruce 2006). In addition, *E. abyssalis* presents a body surface covered with tiny blunt tubercles visible only with scanning electron microscope (SEM) imaging (Pereira and Doti 2017).

In the present contribution, a new species, *Edotia dotiae* sp. nov., is described from the shallow coastal waters of the Argentine Provinces of Chubut, Santa Cruz and Tierra del Fuego – officially the Tierra del Fuego, Antártida e Islas del Atlántico Sur Province. In addition, an identification key to *Edotia* species from South America is provided.

## Material and methods

### Collecting and processing

The sample from Comodoro Rivadavia (Chubut Province) was collected aboard the tugboat “Titan” using a Rauschert sledge equipped with a 1 mm mesh size (2006). Those from Puerto Deseado (Santa Cruz Province) were collected aboard the semi-rigid boat “Los Vikingos” (2006) and the PNA “Río de la Plata” (2007) using a small dredge. Likewise, the specimens from Cabo San Pío (Tierra del Fuego Province) were collected aboard the ARA “Alférez Sobral” employing a small dredge (2002). All specimens were sieved with a 250 μm mesh, fixed with 10% seawater-buffered formalin and transferred to 70% ethanol.

### Dissection and measurements

Appendages were removed under a Leitz stereoscopic microscope employing a sharpened tungsten needle with a 0.137 mm gauge. Measurements were made following Hessler (1970); the apex of the pleotelson was defined and measured according to Pereira et al. (2019). Body lengths were measured in dorsal view from the frontal margin of the head to the tip of the pleotelson. The described specimens were stained with Chlorazol Black E®, and the appendages were temporarily mounted in glycerin.

### Drawings

Drawings of the entire animal were produced using a Leica MZ8 stereoscopic microscope, while drawings of the dissected appendages were made through a Carl Zeiss (Axioskop) compound microscope – both optical devices equipped with a camera lucida. Line drawings were rendered in digital format using the Adobe Illustrator software (Coleman 2003). When relevant, the resulting outlines were printed and stippled by hand, thus avoiding the artifacts reported for digital stippling methods (Martín et al. 2017). These stippled copies were then scanned and composed with their digital contours to produce the final plates.

### Other figures

The light-photographs were taken with a digital microscope Keyence VHX 7000 from the Museum der Nature-Zoologie, Hamburg (Leibniz Institute) in Hamburg, Germany. For SEM photographs, the specimens were cleaned with 0.5% nonionic detergent Triton X100 and ultrasound. After that, they were dehydrated through a graded series of ethanol ending in 100%, critical-point dried, gold-palladium sputter coated, and examined under a Zeiss Gemini SEM 360 microscope at the Museo Argentino de Ciencias Naturales, Buenos Aires, Argentina.

The geographic distribution map was created using the QGIS v.3.34 software (QGIS Development Team 2025), with geographical data downloaded from the Natural Earth repository (Patterson and Vaughn Kelso 2025) and the website of the Instituto Geográfico Nacional de la República Argentina (IGN).

### Institutional repositories

Type and additional material have been deposited in the Invertebrate Collection of the Museo Argentino de Ciencias Naturales “Bernardino Rivadavia” (MACN-In) and in the Carcinological Collection of the Departamento de Biodiversidad y Biología Experimental, Facultad de Ciencias Exactas y Naturales, Universidad de Buenos Aires (DBBE-Cru).

## Results

### Taxonomy

**Idoteidae Samouelle, 1819**

#### Edotia

Taxon classificationAnimaliaValviferaIdoteidae

Guérin-Méneville, 1843

47BC6954-58F6-5D59-A48D-37487275E22D

##### Type species.

*Edotia tuberculata* Guérin-Méneville, 1843 (by monotypy).

##### Diagnosis.

As presented by Brandt and Bruce (2006), except for: Flagellum of antenna rudimentary, composed of 2–4 short articles.

##### Species included.

*E. abyssalis* Pereira & Doti, 2017; *E. acuta* Richardson, 1900; *E. bilobata* Nordenstam, 1933; *E. chilensis* (Nicolet, 1849); *E. corrugata* Sheppard, 1957; *E. dahli* Menzies, 1962; *E. doellojuradoi* Giambiagi, 1925; *E. lilljeborgi* Ohlin, 1901; *E. lyonsi* (Menzies & Kruczynski, 1983); *E. magellanica* Cunningham, 1871; *E. montosa* (Stimpson, 1853); *E. oculata* Ohlin, 1901; *E. oculopetiolata* Sheppard, 1957; *E. pulchra* Brandt, 1990; *E. samariensis* Müller, 1988; *E. sublittoralis* Menzies & Barnard, 1959; *E. tangaroa* Brandt & Bruce, 2006; *E. transversa* Menzies, 1962; *E. triloba* (Say, 1818); *E. tuberculata* Guérin-Méneville, 1843; *E. dotiae* sp. nov.

### Key to *Edotia* species from South America

**Table:** 

1	Pleotelson projected into a long apex; uropodal endopods long, reaching the apex of the pleotelson	***E. dotiae* sp. nov. (Figs 1, 2, 9)**
–	Pleotelson not projected into a long apex; uropodal endopods short	**2**
2	Head without eyestalks, eyes on round lateral lobes or on flat sides of head	**3**
–	Head with eyestalks	**11**
3	Pleotelson dorsomedially with 3 transverse grooves which do not reach the lateral sides of pleotelson	***E. tuberculata***
–	Pleotelson without dorsomedial transverse grooves	**4**
4	Pleotelson with at least 1 complete suture	**5**
–	Pleotelson without complete sutures	**8**
5	Pleotelson with 1 complete suture	**6**
–	Pleotelson with 2 complete sutures	**7**
6	Head, frontal margin with 2 large, rounded lobes between antennae	***E. bilobata***
–	Head, frontal margin without 2 large, rounded lobes between antennae	***E. chilensis***
7	Pleotelson oval, distally concave	***E. doellojuradoi***
–	Pleotelson subtriangular, distally acuminate	***E. transversa***
8	Pleotelson with pleonite 1 visible as lateral epimera	***E. magellanica***
–	Pleotelson without visible lateral epimera	**9**
9	Pleotelson pentagonal, lateral margins straight	***E. lilljeborgi***
–	Pleotelson lateral margins convex	**10**
10	Head with 1 dorsal rounded protuberance; frontal lamina not visible in dorsal view	***E. corrugata***
–	Head with 1 dorsal bilobated protuberance; frontal lamina projected anteriorly, visible in dorsal view	***E. dahli***
11	Eyestalks with ommatidia; dorsal surface of pereonites 1–5 smooth	***E. oculata***
–	Eyestalks without ommatidia; dorsal surface of pereonites 1–5 with 1 transverse ridge	***E. abyssalis***

#### Edotia dotiae sp. nov.

Taxon classificationAnimaliaValviferaIdoteidae

502D9753-3EE6-55F7-8F2D-1F9A217933BB

https://zoobank.org/A423E1CC-AA0D-47EC-82DC-ECFAE771F05E

Figs 1, 2, 3, 4, 5, 6, 7, 8, 9

##### Synonymy.

*Edotia* n. sp. “A” Pereira 2017: 61, fig. 30.

##### Material examined.

***Holotype***: Argentina – **Santa Cruz Province, Puerto Deseado** • 1 brooding ♀ (4.3 mm); 47°43.76'S, 65°50.26'W, 15 m depth; 23 Jan. 2007; I. Chiesa, B. Doti, D. Roccatagliata leg.; Sta. 20, small dredge, PNA Río de la Plata; MACN-In 44878.

***Paratypes***: Argentina • 1 non-brooding ♀ (4.4 mm), 4 mancas and juvs (1.3–2.8 mm), same data as for holotype; MACN-In 44879. – **Santa Cruz Province, Puerto Deseado** • 1 adult ♂ (4.7 mm), 2 non-brooding ♀♀ (4.0–4.4 mm); 47°45.42'S, 65°52.63'W, 2–15 m depth; 7 Feb. 2006; R. Centurión, I. Chiesa, B. Doti, C. Muniain, D. Roccatagliata leg.; Sta. 11, small dredge, semi-rigid boat Los Vikingos; MACN-In 44880.

##### Additional material.

Argentina – **Santa Cruz Province, Puerto Deseado** • 1 manca (1.6 mm); 47°48.89'S, 65°51.25'W, 15 m depth; 23 Jan. 2007; I. Chiesa, B. Doti, D. Roccatagliata leg.; Sta. 15, small dredge, PNA Río de la Plata; DBBE-Cru 1581. • 1 non-brooding ♀ (4.0 mm), 3 mancas (2.3–2.9 mm); 47°45.41'S, 65°52.64'W, 2–15 m depth; 07 Feb. 2006; R. Centurión, I. Chiesa, B. Doti, C. Muniain, D. Roccatagliata; Sta. 12, small dredge, semi-rigid boat Los Vikingos; DBBE-Cru 1582. • 1 adult ♂ (4.0 mm), 2 mancas (1.7–3.0 mm); 47°45.42'S, 65°52.60'W, 2–15 m depth; 7 Feb. 2006; R. Centurión, I. Chiesa, B. Doti, C. Muniain, D. Roccatagliata leg.; Sta. 13, small dredge, semi-rigid boat Los Vikingos; DBBE-Cru 1583. – **Chubut Province, Comodoro Rivadavia** • 1 adult ♂ (4.2 mm), 1 manca (2.3 mm); 45°51.36'S, 67°27.13'W, 13.8 m depth; 5 Feb. 2006; R. Centurión, I. Chiesa, B. Doti, C. Muniain, D. Roccatagliata leg.; Sta. 6, Rauschert sledge, tugboat Titan; DBBE-Cru 1584. – **Tierra del Fuego Province, Cabo San Pío** • 6 mancas and juvs (2.6–3.0 mm); 55°03.00'S, 67°37.00'W, 30–35 m depth; 29 Sep. 2002; D. Zelaya leg.; Sta. SP, small dredge, ARA Alférez Sobral; DBBE-Cru 1585.

##### Diagnosis.

Body with several discrete patches of fluted tubercles. Head with frontal lamina triangular, with 2 anterodorsal lobes. Pleonite 1 indicated by lateral epimera. Pleotelson lateral margins concave, distally protruding into a long apex (apex 0.3 times as long as pleon). Flagellum of antenna composed of 4 short articles. Endopod of uropod elongated, reaching the tip of the pleotelson apex.

##### Description.

Body description is based on the holotype female (MACN-In 44878) and the appendages on the paratype female (MACN-In 44879-a).

**Figure 1.**
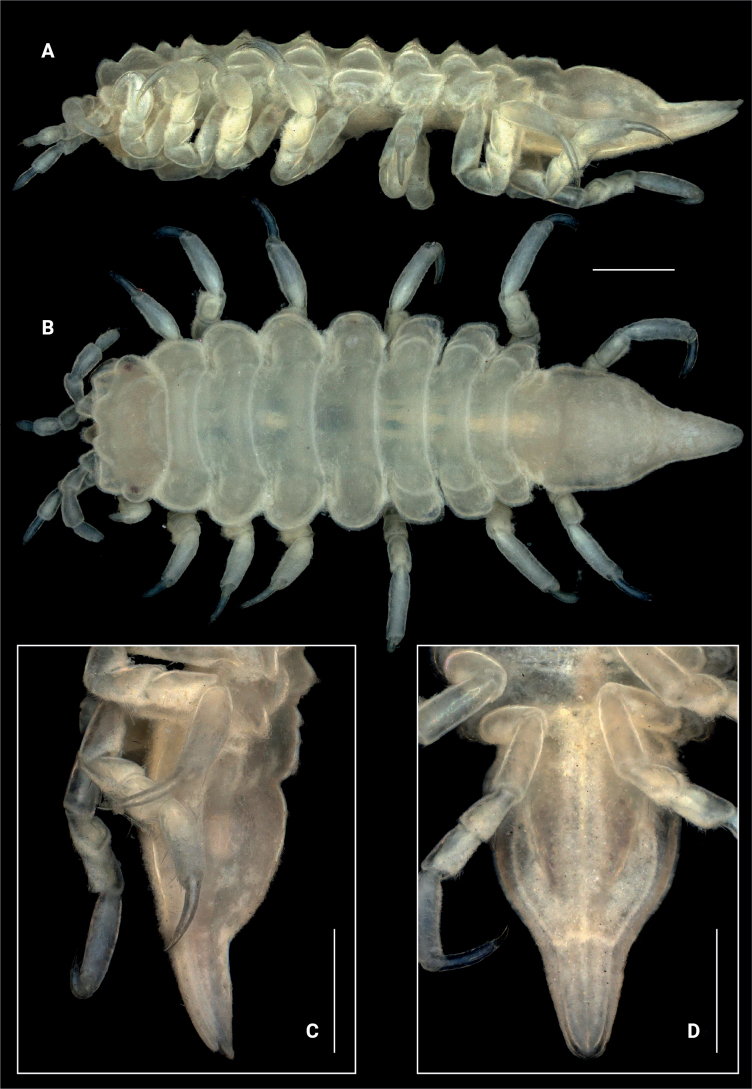
*Edotia dotiae* sp. nov., paratype female (MACN-In 44880-b). **A, B**. Habitus in lateral and dorsal view, respectively; **C**. Pleotelson in lateral view; **D**. Pleotelson in ventral view. Scale bars: 0.5 mm.

**Figure 2.**
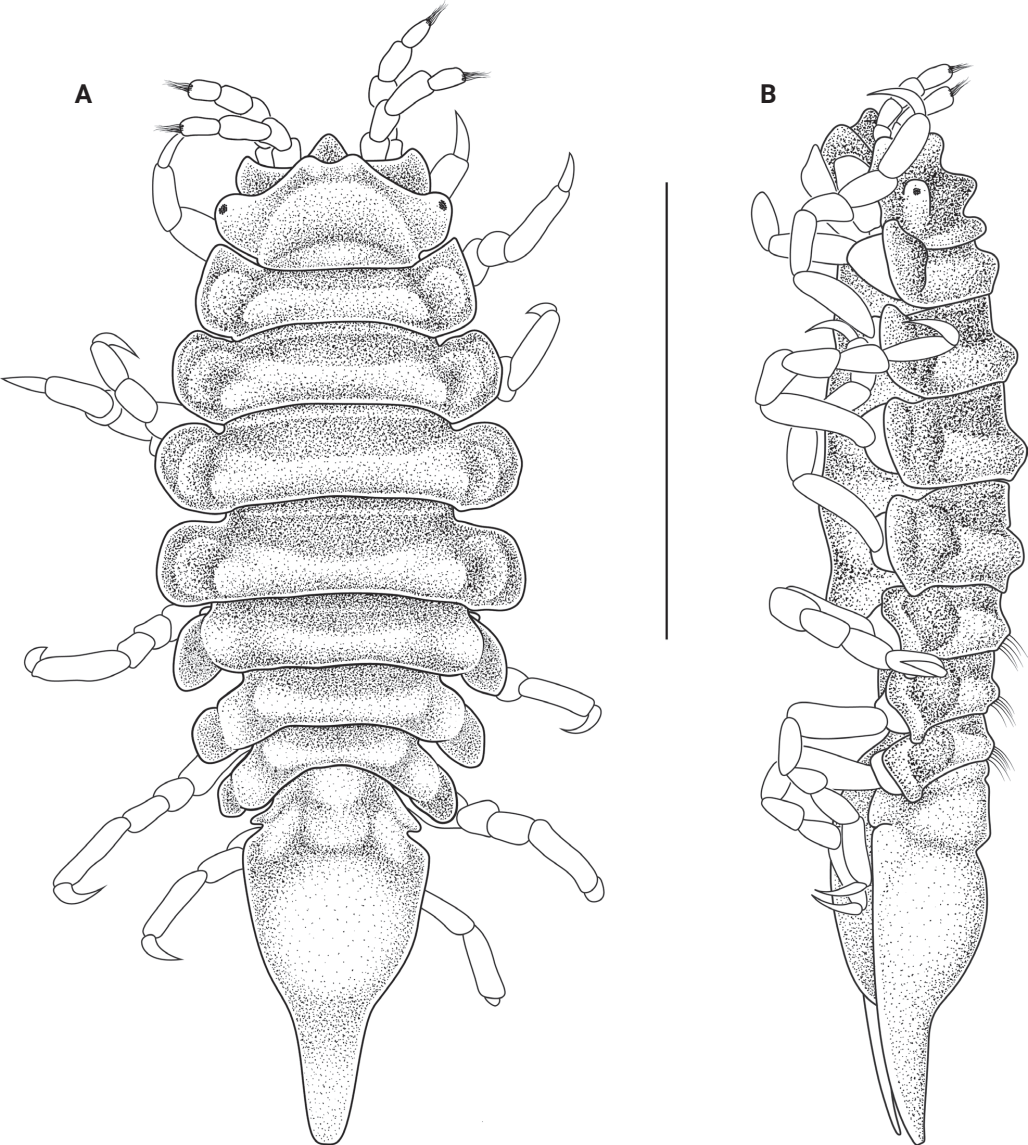
*Edotia dotiae* sp. nov., holotype female (MACN-In 44878). **A**. Habitus in dorsal view; **B**. Habitus in lateral view. Scale bars: 2 mm.

***Body*** (Figs 1, 2, 9) flattened, widest at pereonites 3 and 4. ***Body surface*** and ***lateral margins*** covered with short setae and discrete patches of fluted tubercles (Fig. 9A–D). ***Head*** length 0.5 width, with 1 wide middorsal elevation ornamented with 2 patches of fluted tubercles, and 2 anterodorsal lobes with fluted tubercles; lateral margins rounded, with small eyes; frontal margin straight, with 2 pointed lateral projections. ***Frontal lamina*** triangular, visible in dorsal view. ***Pereonite*** 1 similar in length to pereonites 5–7; pereonites 2 and 3 subequal in length; pereonite 4 longest; pereonites 5–7 becoming progressively shorter and narrower. ***Dorsal surface*** of pereonites 1–7 with a shallow carina close to posterior margin, covered with fluted tubercles. ***Lateral margin*** of pereonite 1 with square anterolateral angle; pereonites 2–7 rounded; directed anteriorly in pereonites 1–3, laterally in pereonite 4, and posteriorly on pereonites 5–7. ***All pleonites*** fused with the pleotelson; pleonite 1 indicated by lateral epimera. ***Pleotelson*** lateral margins concave, distally protruding into a long apex (apex 0.3 times as long as pleon).

**Figure 3.**
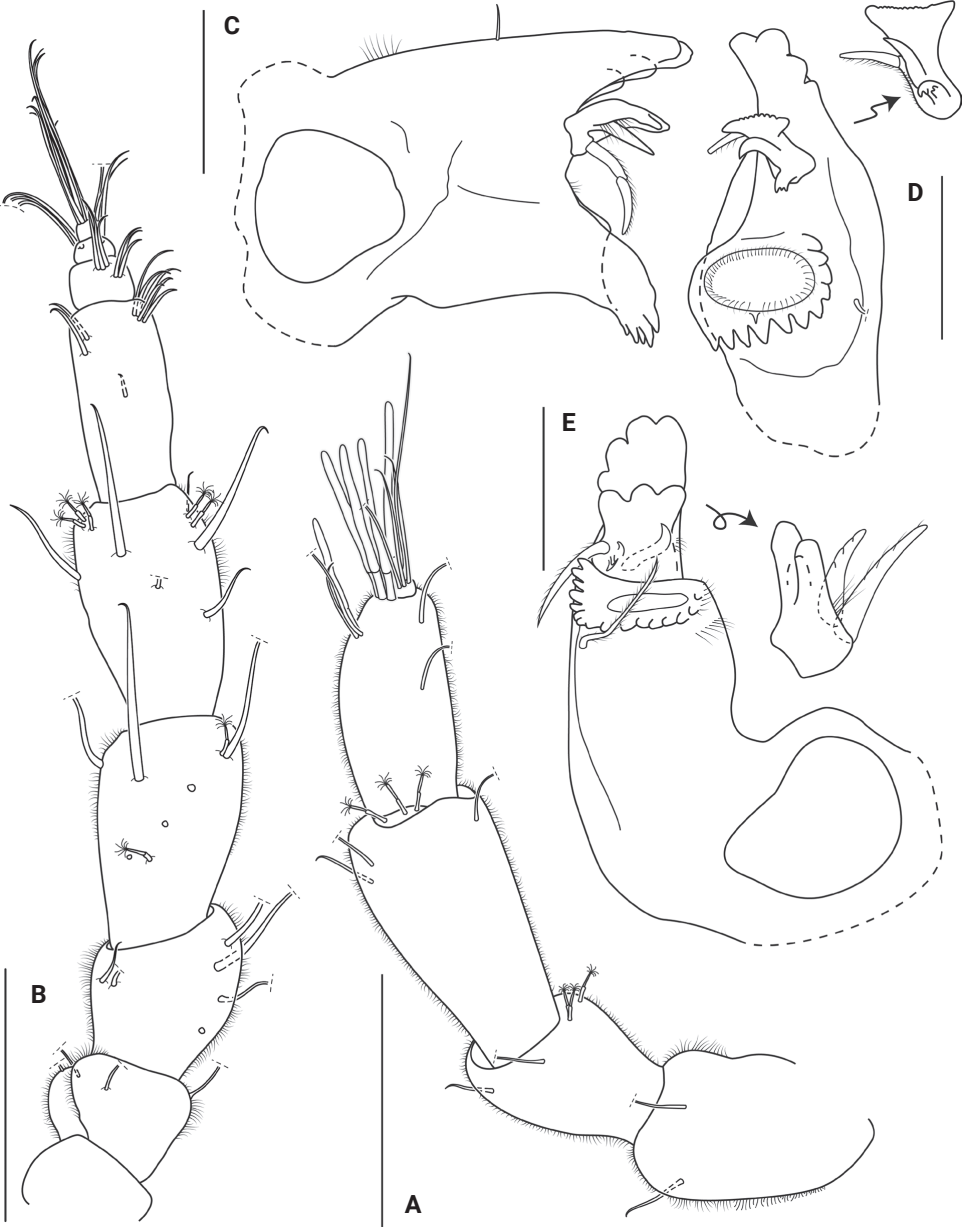
*Edotia dotiae* sp. nov., paratype female (MACN-In 44879-a). **A**. Right antennula; **B**. Right antenna; **C, D**. Left mandible in different views, with detail of *lacinia mobilis* and spine row; **E**. Right mandible with detail of molar process. Scale bars: 0.2 mm.

***Antennula*** (Fig. 3A) with 3 peduncular and 3 flagellar articles; peduncular article 1 similar in length to flagellar article 2, with 2 microsetulate setae; article 2 0.6 times as long as article 3, with 3 broom and 2 microsetulate setae; article 3 longest, with 3 microsetulate setae. Flagellar article 1 forming a short ring, with 3 broom setae; article 2 longest, with 4 aesthetascs and 4 microsetulate setae; last article smallest, knob-like, with 1 aesthetasc and 4 microsetulate setae. All articles covered with short setae.

***Antenna*** (Fig. 3B) with 5 peduncular and 4 flagellar articles; first two peduncular articles 0.5 times as long as article 5, article 1 glabrous, article 2 with 2–4 microsetulate setae; article 3 0.7 times as long as article 5, with 3–6 microsetulate setae; article 4 subequal in length to article 5, with 0–2 broom and 4–6 microsetulate setae; article 5 longest, with 5 broom and 6 microsetulate setae. Flagellar article 1 longest article, with 9 microsetulate setae; article 2 and 3 less than 0.3 times as long as article 1, with 6 microsetulate setae each; last article smallest, knob-like, with 8 microsetulate setae. All articles covered with short setae.

***Mandibles*** (Fig. 3C–E) asymmetrical, without palp. Incisor processes with 4 strong sclerotized teeth. Molar processes with grinding surface and indented margins, with 1 seta on lower surface. Left ***lacinia mobilis*** with 3 rounded teeth and 2 stout setae; right ***lacinia mobilis*** bifid, apically serrated; spine row comprising 3 seta-like structures.

**Figure 4.**
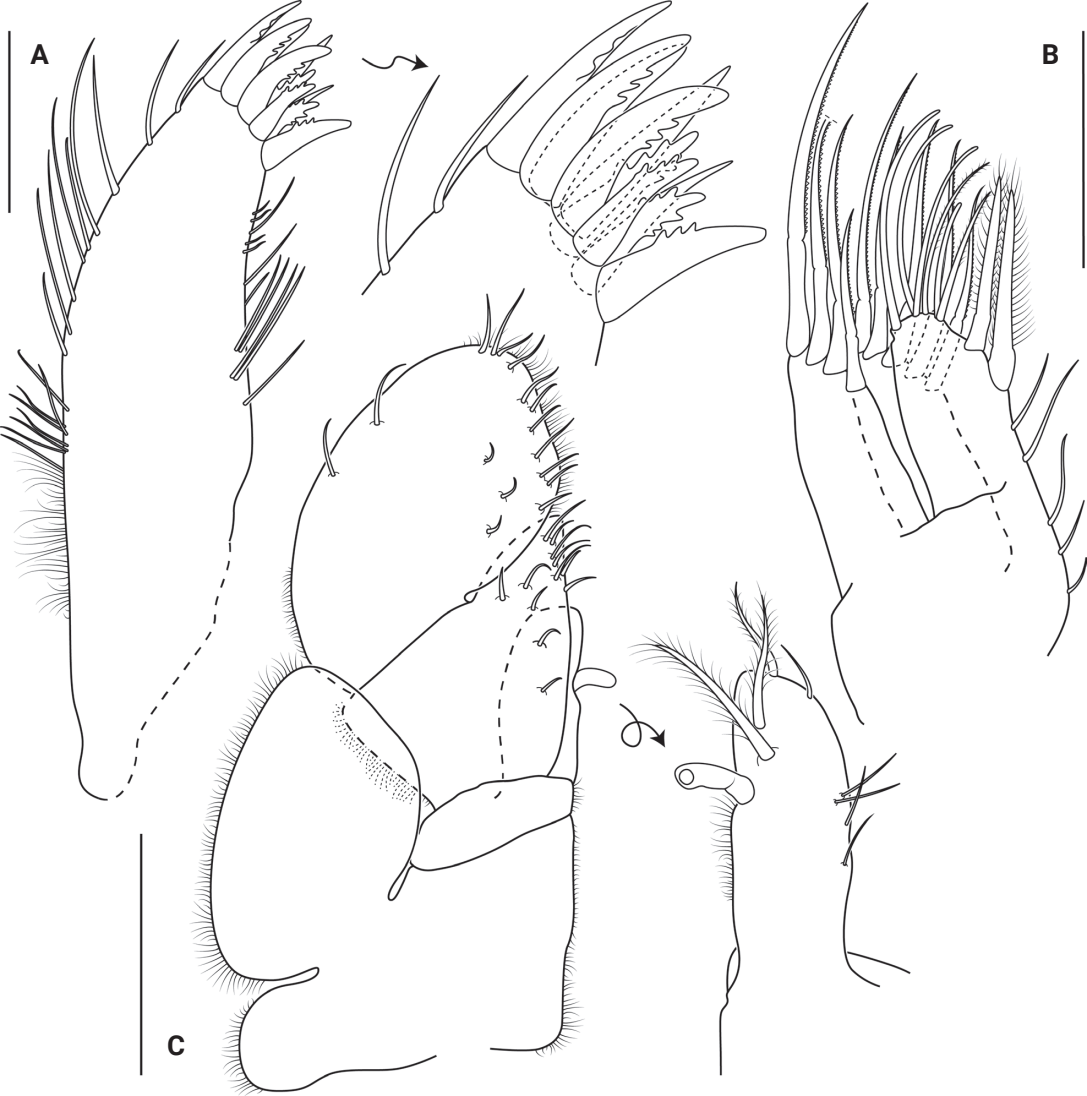
*Edotia dotiae* sp. nov., paratype female (MACN-In 44879-a). **A**. Right maxillula; **B**. Right maxilla; **C**. Right maxilliped in posterior view, with detail of endite in anterior view. Scale bars: 0.2 mm.

***Maxillula*** (Fig. 4A) lateral lobe with 10 stout setae distally, some of them serrated, with simple and microsetulate setae laterally. Inner lobe missing in dissection.

***Maxilla*** (Fig. 4B) outer lobe with 4 serrate setae distally. Middle lobe with 5 serrate setae distally. Inner lobe with 2 stout setulated setae, 2 thin distally setulated setae and 4 simple setae rounded tip. Inner margin with 5 simple setae.

***Maxiliped*** (Fig. 4C) palp with 3 articles; article 1 shortest; article 2 trapezoidal with 12 microsetulate setae; article 3 largest, distal margin convex, with 16 microsetulate setae. Epipod oval, narrowing distally, reaching proximal margin of palp article 3. Basal endite slender, anterior surface with 3 plumose setae distally, outer margin with 5 microsetulate setae, mesial margin with 1 coupling seta. All maxilliped covered with setules.

**Figure 5.**
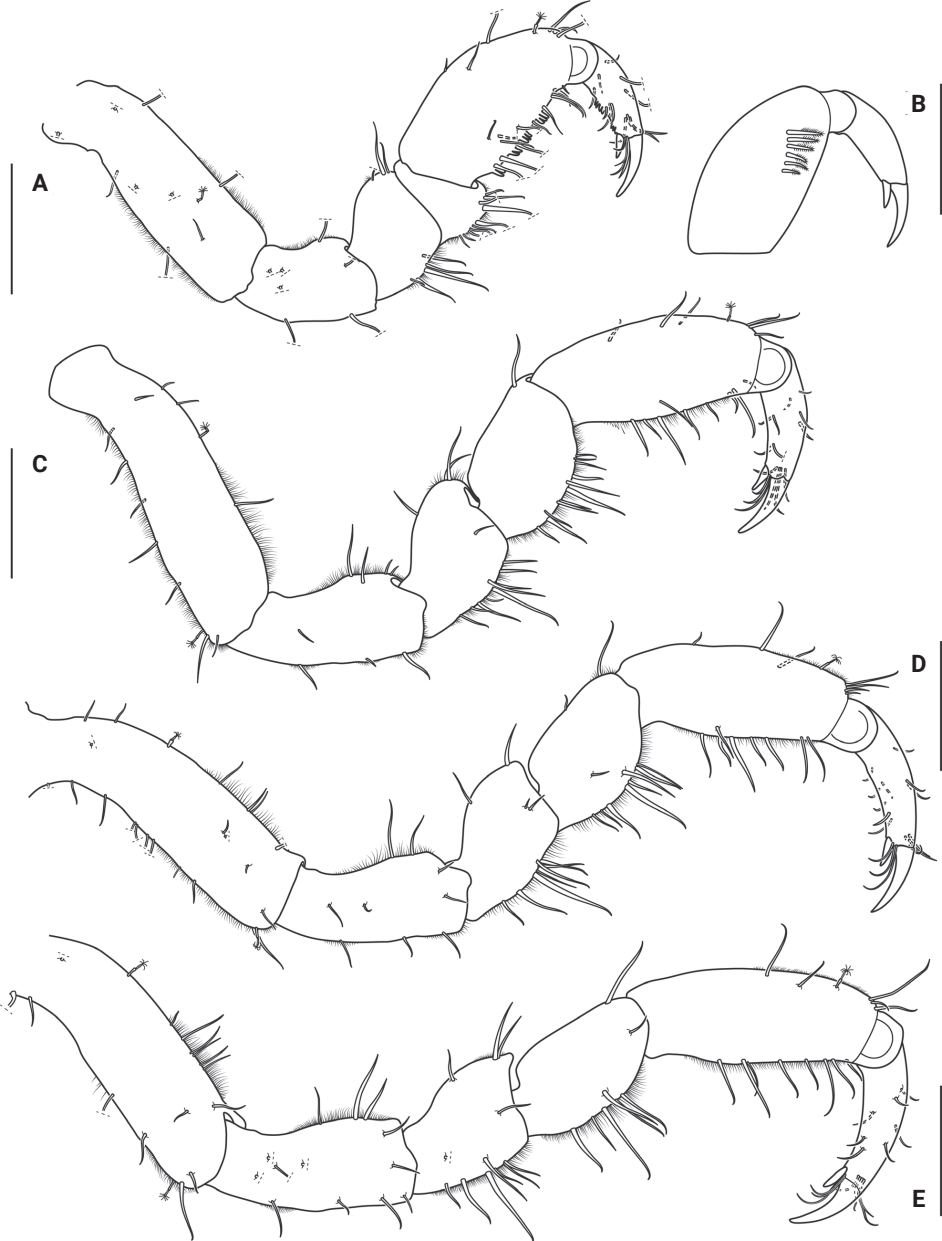
*Edotia dotiae* sp. nov., paratype female (MACN-In 44879-a). **A**. Right pereopod I; **B**. Robust biserrate setae on inner surface of left pereopod I; **C–E**. Right pereopods II–IV, respectively. Scale bars: 0.2 mm.

***Pereopod I*** (Figs 5A, 5B, 10A, 10B) subchelate, basis longest article, with 1 or 2 broom and 9 or 10 microsetulate setae; ischium 0.6 times as long as propodus, with 7 microsetulate setae; merus 0.4 times as long as propodus, with 8 or 9 microsetulate setae; carpus shortest article, with 10 microsetulate setae on flexor margin; propodus oval, with 9 microsetulate setae and cutting teeth on flexor margin, 4 microsetulate setae close to flexor margin, 1 broom and 6 microsetulate setae on extensor margin; with 6 robust biserrate setae on inner surface; dactylus (excluding claws) 0.5 times as long as propodus, with cutting teeth on flexor margin and several microsetulate setae on both margins.

***Pereopods II–IV*** (Fig. 5C–E) basis longest article, with 2 broom and 11–20 microsetulate setae; ischium 0.8 times as long as propodus, with 8–13 microsetulate setae; merus 0.5 times as long as propodus, with 11–13 microsetulate setae; carpus 0.5–0.6 times as long as propodus, with 8–12 microsetulate setae; propodus oval, with 1 broom seta dorsodistally, 5–7 microsetulate setae on extensor margin, 8 or 9 microsetulate setae on flexor margin; dactylus (excluding claws) 0.4–0.6 times as long as propodus, with 2 distal claws, secondary unguis 0.4 times as long as primary unguis, with several microsetulate setae on both margins.

**Figure 6.**
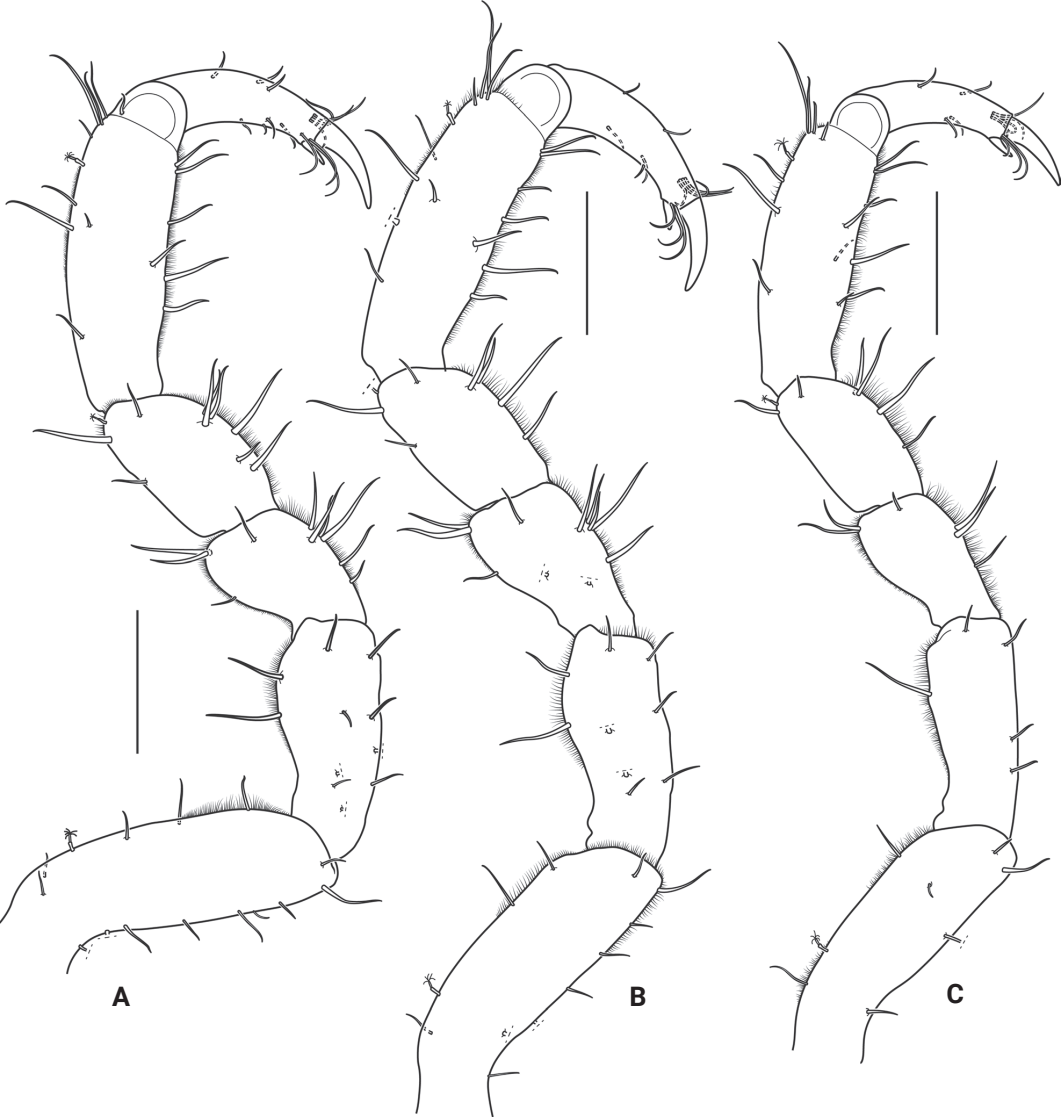
*Edotia dotiae* sp. nov., paratype female (MACN-In 44879-a). **A–C**. Right pereopods V–VII, respectively. Scale bars: 0.2 mm.

***Pereopods V–VII*** (Fig. 6A–C) basis longest article, with 1 broom and 7–14 microsetulate setae; ischium 0.7 times as long as propodus, with 5–11 microsetulate setae; merus 0.4–0.5 times as long as propodus, with 6–11 microsetulate setae; carpus 0.5 times as long as propodus, with 1 broom and 6–9 microsetulate setae; propodus oval, with 1 broom seta distally on extensor margin, with 7–9 microsetulate setae on extensor margin and 6–8 microsetulate setae on flexor margin; dactylus (excluding claws) 0.5–0.6 times as long as propodus, with 2 distal claws, secondary unguis 0.3 times as long as primary unguis, with several microsetulate setae on both margins.

**Figure 7.**
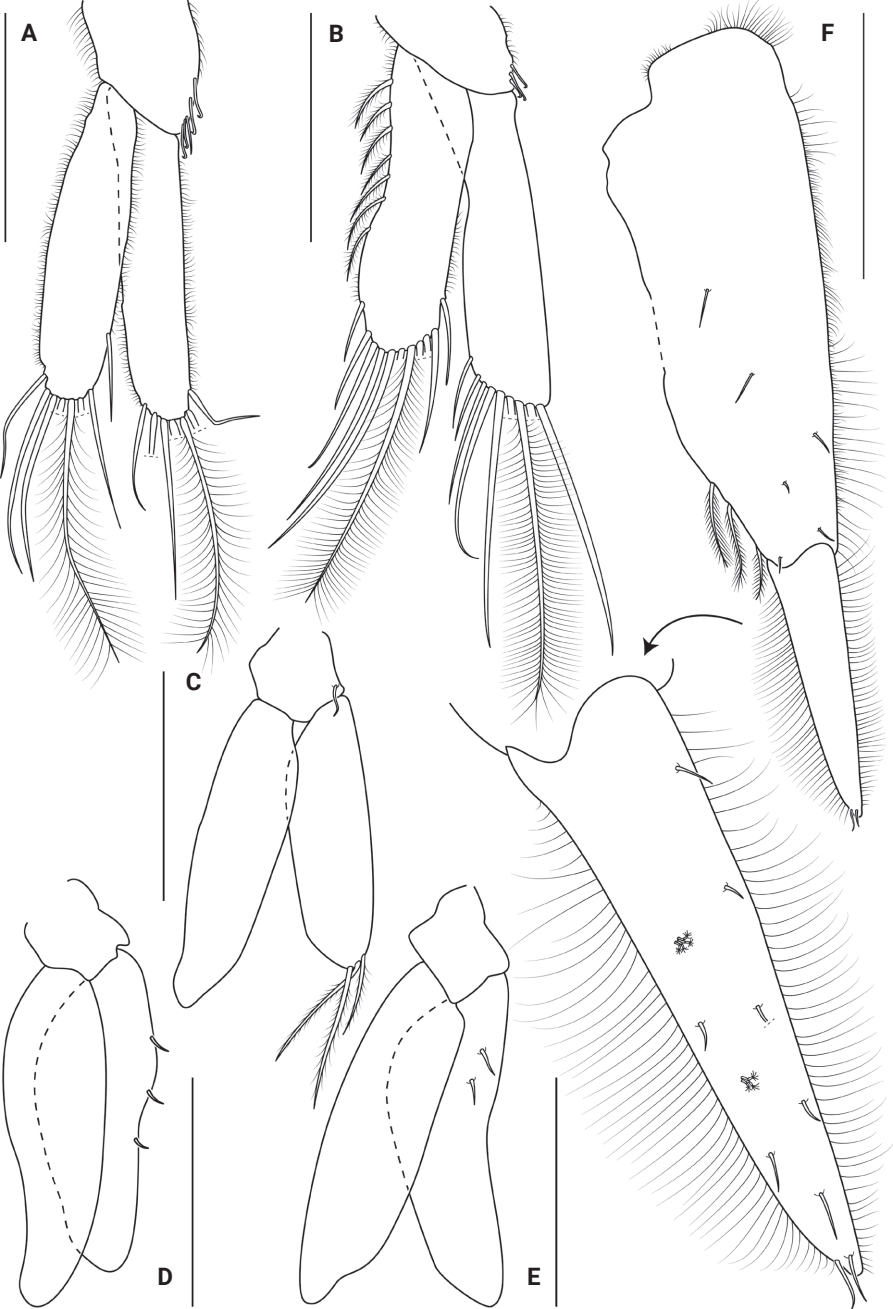
*Edotia dotiae* sp. nov., paratype female (MACN-In 44879-a). **A–E**. Right pleopods I–V, respectively; **F**. Right uropod, with detail of endopod. Scale bars: 0.3 mm (**A–E**); 1 mm (**F**).

***Pleopod I*** (Fig. 7A) protopod quadrangular, with 5 coupling setae on inner margin. Endopod subequal in length to exopod, with 7 plumose setae on distal margin; exopod with 1 plumose seta on inner margin and 7 plumose setae on distal margin. Both rami with setules on lateral margins.

***Pleopod II*** (Fig. 7B) protopod quadrangular, with 3 coupling setae on inner margin. Endopod subequal in length to exopod, with 10 plumose setae on distal margin; exopod with 7 plumose setae on lateral margin, 11 plumose setae on distal margin, and setules on both margins.

***Pleopod III*** (Fig. 7C) protopod quadrangular, with 1 simple seta. Endopod subequal in length to exopod, with 0 or 1 simple seta and 2 or 3 plumose setae on distal margin. Exopod glabrous.

***Pleopods IV and V*** (Fig. 7D, E) protopod quadrangular, glabrous. Endopod subequal in length to exopod, with 2 or 3 microsetulate setae on lateral margin. Exopod glabrous.

***Uropod*** (Fig. 7F) uniramous, reaching to pleotelson apex. Protopod, mesial surface with 6 microsetulate setae, 2 or 3 plumose setae on inner margin, and setules on outer margin. Endopod 0.5 times as long as protopod, mesial surface with 6 broom setae and 5 or 6 microsetulate setae, 2 microsetulate setae on distal tip, and both margins setulated.

**Figure 8.**
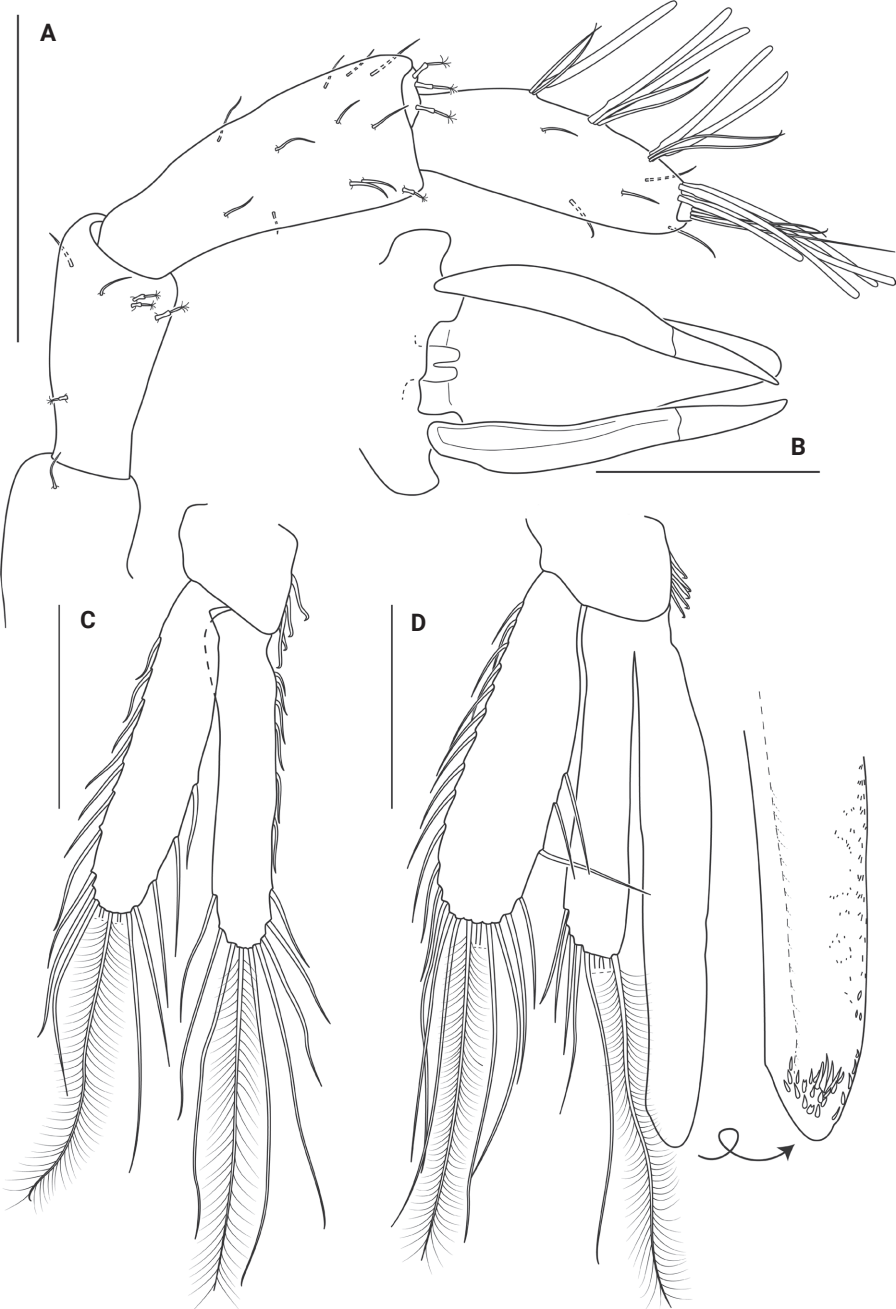
*Edotia dotiae* sp. nov., paratype male (MACN-In 44880-a). **A**. Right antennula; **B**. Pleon in ventral view, with open uropods; **C**. Right pleopod; **D**. Right pleopod II with detail of *appendix masculina*. Scale bars: 0.3 mm (**A, C, D**); 1 mm (**B**).

**Paratype male** (MACN-In 44880-a) as female except for:

***Antennula*** (Fig. 8A) with 3 peduncular and 3 flagellar articles; peduncular article 1 about 0.5 times as long as article 3, with 1 or 2 microsetulate setae; article 2 0.6 times as long as article 3, with 4 broom and 2 microsetulate setae; article 3 longest, with 1 broom and 11 microsetulate setae. Flagellar article 1 forming a short ring, with 3 broom setae; article 2 0.8 times as long as peduncular article 3, with 9 or 10 aesthetascs and 5 microsetulate setae; last article smallest, knob-like, with 3 microsetulate setae. All articles covered with short setae.

**Figure 9.**
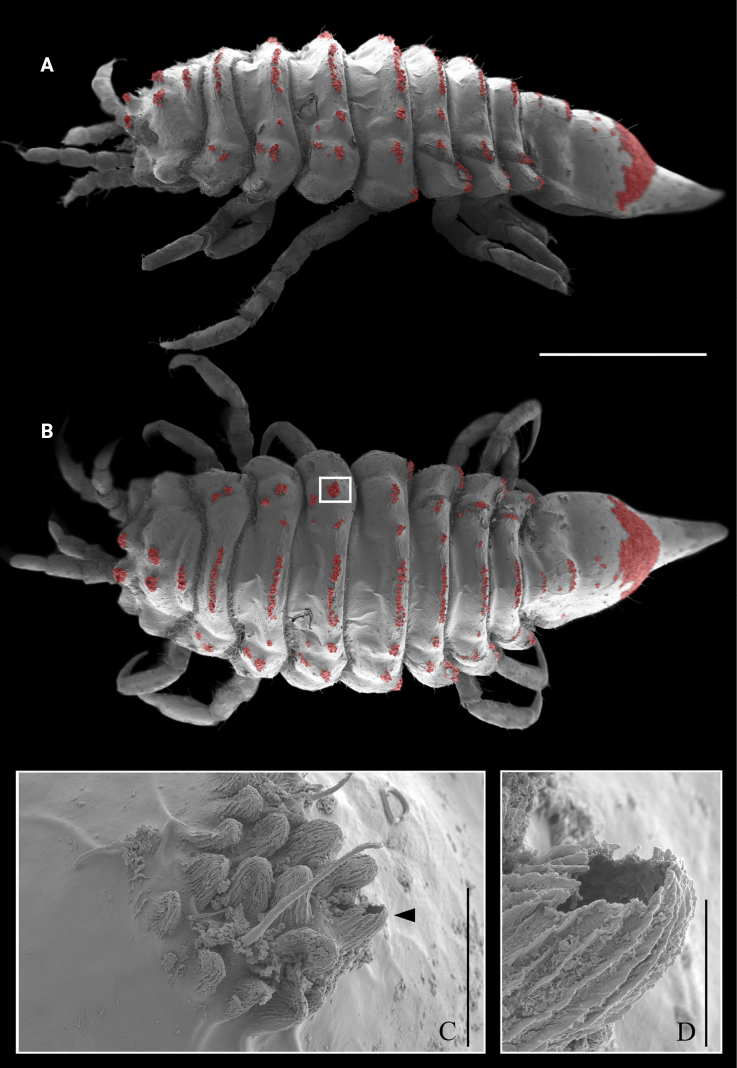
*Edotia dotiae* sp. nov., SEM photographs, paratype female (MACN-In 44880-c). **A, B**. Habitus in lateral and dorsal view, respectively; red areas indicate patches of fluted tubercles; **C**. Detail of a patch of fluted tubercles; **D**. Detail of a fluted tubercle, showing the hollow interior. Scale bars: 1 mm (**A, B**); 0.05 mm (**C**); 0.01 mm (**D**).

**Figure 10.**
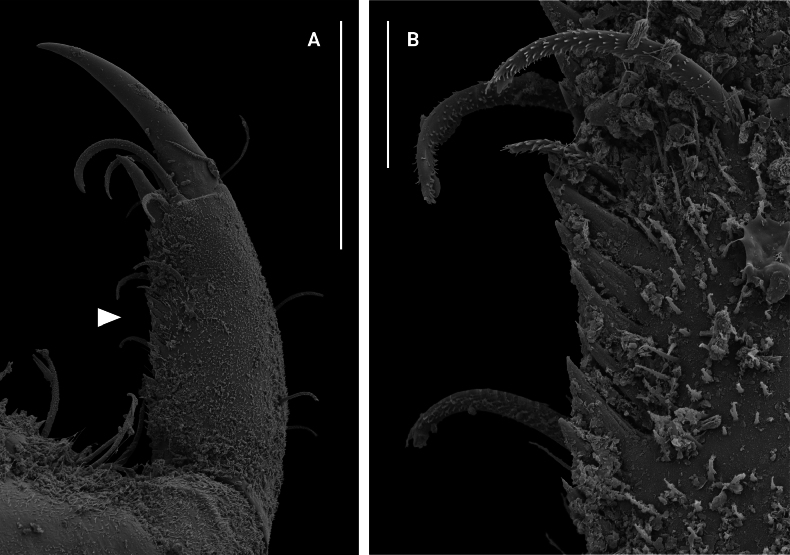
*Edotia dotiae* sp. nov., SEM photographs, paratype female (MACN-In 44880-c). **A**. Dactylus of pereopod I; **B**. Detail of cutting teeth on flexor margin of dactylus of pereopod I. Scale bars: 0.1 mm.

***Penes*** (Fig. 8B) fused basally as a penial plate but divided over most length.

***Pleopod I*** (Fig. 8C) protopod quadrangular, with 4 coupling setae on inner margin. Endopod subequal in length to exopod, with 6 plumose setae on inner margin and 9 plumose setae on distal margin. Exopod with 3 plumose setae on inner margin, 8 plumose setae on distal margin and 7 plumose setae on outer margin. Both rami with setules on lateral margins.

***Pleopod II*** (Fig. 8D) protopod quadrangular, with 5 coupling setae on inner margin. Endopod subequal in length to exopod, with 8 plumose setae on distal margin. ***Appendix masculina*** 1.5 times as long as endopod, with several spines subapically. Exopod with 3 plumose setae on inner margin, 11 plumose setae on distal margin and 10 plumose setae on outer margin. Both rami with setules on lateral margins.

##### Etymology.

The newly described species is named after Brenda L. Doti, our mentor and colleague, in recognition of her significant and growing contribution to the knowledge of marine isopods from Argentina.

##### Distribution.

**Figure 11.**
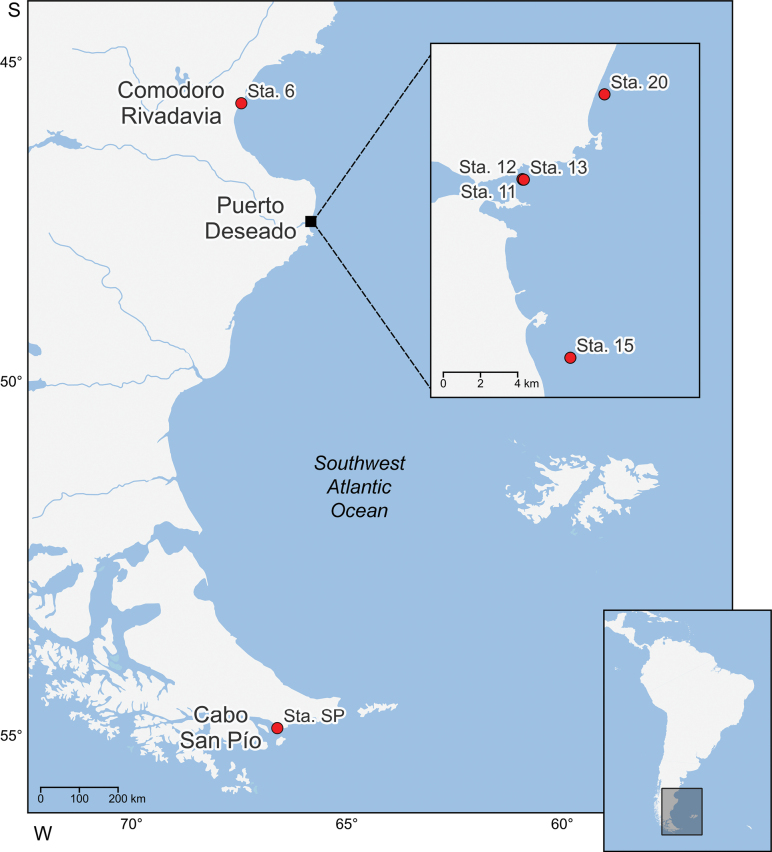
Geographic distribution of *Edotia dotiae* sp. nov. along southern part of Argentina.

From the coast of Chubut Province to the Beagle Channel, in a depth range of 2–35 m (Fig. 11).

##### Remarks.

*Edotia dotiae* sp. nov. can be distinguished from other *Edotia* species by the long uropodal endopod, which reaches the tip of the long apex of the pleotelson. This arrangement is unique within the genus.

Aside from the above, the general aspect of *Edotia dotiae* sp. nov. broadly resembles that of *E. lyonsi* and *E. samariensis*, but the three species differ in the shape of their frontal laminae – which is triangular in *E. dotiae* sp. nov. as opposed to bifid in *E. lyonsi* and rounded in *E. samariensis*. They also differ in the lateral epimera of the first pleonite – which are acute and subtle in *E. dotiae* sp. nov. as opposed to rounded and broad in both *E. lyonsi* and *E. samariensis*.

## Discussion

*Edotia dotiae* sp. nov. is the only species of the family Idoteidae in which the pleotelson projects distally in a long apex while simultaneously the endopod of the uropod is elongated about 0.5 times the length of the protopod and reaches the tip of the apex. Although some species satisfy each of these conditions independently, the combination is rare even within the suborder Valvifera. The idoteid species *Crabyzos longicaudatus* Bate, 1863 and *Euidotea caeruleotincta* Hale, 1927, for example, have the pleotelson projected distally in a long apex, but do not have elongated uropodal endopods (0.2 times as long as the protopod for *C. longicaudatus* and 0.4 for *E. caeruleotincta*), and these do not reach the tip of the pleotelson (Poore and Lew Ton 1993). On the other hand, the species *Paridotea collingei* Poore & Lew Ton, 1993 and *P. simplex* Poore & Lew Ton, 1993 have uropodal endopods that reach the tip of the pleotelson but are not elongated (0.3 times as long as protopod in both species; Poore and Lew Ton 1993); furthermore, even though both species have a long pleotelson, these do not project distally in a long apex as defined by Pereira et al. (2019). Within the suborder Valvifera, only the species of the genus *Xiphoarcturus* Pereira, Roccatagliata & Doti, 2019 share this combination of characters with *Edotia dotiae* sp. nov.

Most species of the family Idoteidae present a relatively smooth body surface, without spines or significant cuticular structures. In particular, within the genus *Edotia*, among the 20 previously described species, only three show some ornamentation on the body surface: *E. pulchra* presents two conical tubercles on the head, dorsal carinae on pereonites, and small tubercles on the pleotelson (Brandt 1990); *E. tangaroa* shows tubercles almost cauliflower-shaped on the head and dorsal surface with small blunt tubercles (Brandt and Bruce 2006); and the third species, *E. abyssalis*, also presents high dorsal carinae on pereonites 1–5 (Pereira and Doti 2017). Additionally, the examination of the latter species under the SEM demonstrated that the body surface is also covered with tiny blunt tubercles and short setae (Pereira and Doti 2017, fig. 7). The new species, *Edotia dotiae* sp. nov., shows shallow carinae on the body surface, and the SEM examination revealed the presence of patches of fluted tubercles on the dorsal body surface (Fig. 9). High-magnification imaging (e.g. SEM) has rarely been used in the description of *Edotia* species (Pereira and Doti 2017, present contribution), but in those cases the presence of tiny cuticular structures has been noticed. This suggests that other *Edotia* species may also possess some kind of cuticular structures that were previously overlooked, such as tiny tubercles or numerous short setae. Furthermore, studying the microanatomy of these structures could provide new insights into their functionality and the biology of these isopods.

Regarding their geographic distribution, among the 20 previously described *Edotia* species, six are present in the Northern Hemisphere: from Caribbean Sea to the western North Atlantic, and in eastern coast of the North Pacific (Say 1818; Stimpson 1853; Richardson 1900; 1905, Menzies and Barnard 1959; Schultz 1969; Goeke and Heard 1983; Menzies and Kruczynski 1983; Müller 1988; Rafi and Laubitz 1990; Stebbins and Wetzer 2023; among others); other three species are reported from the Subantarctic and Antarctic waters (Sheppard 1957; Brandt 1990; Castelló 2004; Brandt and Bruce 2006); whereas the remaining 11 species are recorded from both coasts of South America’s Southern Cone (Guérin-Méneville 1843; Nicolet 1849; Cunningham 1871; Ohlin 1901; Giambiagi 1925; Nordenstam 1933; Sheppard 1957; Menzies 1962; Pereira and Doti 2017; among others). *Edotia dotiae* sp. nov. is recorded from the Atlantic sector of Patagonia and the Beagle Channel. The finding of a new species in the Southwest Atlantic supports the fact that the diversity of the genus is particularly high in this region. The reasons for this asymmetrical geographic distribution remain elusive, and biogeographical and phylogenetic studies could be conducted to better understand the evolutionary history of the genus *Edotia*.

## Supplementary Material

XML Treatment for Edotia

XML Treatment for Edotia dotiae

## Additional information

### Use of AI

No use of AI was reported.

### Author ORCIDs

Julián Santiago https://orcid.org/0009-0000-9502-4387

Emanuel Pereira https://orcid.org/0000-0001-5495-7148

### Data availability

All of the data that support the findings of this study are available in the main text.
